# Influence of Variola and Vaccinia

**Published:** 1854-10

**Authors:** W. Cooper


INFLUENCE OF VARIOLA AND VACCINIA. By W. COOPER, ESQ., M. R. C. S. E.—Through tbe kindness of Mr. Skegg, of St. Martin's-place, I have seen, in the Strand, a case completely illustrative of the mutual influence of variola and vaccinia.

A girl, exhibiting no vaccine cicatrix, had attended and slept in the same room with her sister, affected with confluent variola. During this exposure she was vaccinated by Mr. Skegg, on the 18th of May, with fresh lymph. Three punctures were perfect. On the 22nd, premonitory symptoms of variola were observed; and on the 26th, papulæ. This was the eleventh day from the vaccination. On the 30th, when I saw her there were three very large, round, flat, dull-yellow pustules at the points of vaccination; variola plentifully scattered over the body. On the 2nd of June, eighteen days from the punctures, and seven days from the variolous papulæ, the variolous pustules changed to a brown, filmy scale, rapidly falling off, nothing like maturation or secondary fever occurring.

The points both of curiosity and practical interest are, the synchronously modified or hybrid character of the two pocks, seeming, unless variola and vaccine be identical, to invalidate John Hunter's axiom; the antidotal or extreme mitigation of semi-confluent and extensive variola, although not absolutely prophylactic, and, above all, the total prevention of maturation and secondary fever. *A priori*, we should at once pronounce, on the seventh day of a perfect areolar and progressive vaccine vesicle, complete immunity from variola; nor should we be prepared to see, as in this case, vesicles converted into unilocular cysts containing discs like the matured variolous pustule. A slight revolution has taken place regarding Jenner's adaption. Such a case as this, however, should make the objector pause, as it proves the value of vaccine far more than cases of prophylaxis, which may often be merely negative. These cases are very rare, but I could refer to five or six others. My point, however, would not be thereby strengthened.—*London Lancet*, June, 1854.

Calomel and Quinine in Tropical Fever.—There cannot be a doubt that if not calomel, yet certainly salivation, is an antidote to malarious fever. The instant a patient's mouth is sore, the fever leaves him. The mercury produces not the slightest effect till then ; but from that moment the disease vanishes as if charmed. The change is from death to life—from extremity of suffering to calm and comfort. Numerous instances, too, of the safety which salivation gives from the effects of the malarious poison, may be found in Dr. Johnson's book, such as patients salivated for syphilis sleeping with impunity in places which were fatal to every one of their companions ; and also many cases are on record of officers in India passing in a state of salivation by Dâk, unharmed, through the most deadly jungles. But since the discovery of the invaluable quinine, we have wanted a bold return to the practice of our forefathers ; for in quinine we have all the qualities required. It is not a bulky medicine, and does not cause vomiting. It is a perfect antidote, and will cure all cases of malarious fever not otherwise hopeless, and it has no bad effect, like mercury, on the constitution. The gradual introduction of quinine in place of calomel has left our mortality by fever much the same as in former days ; but by the expectant treatment for malarious dysentery, where quinine has not been substituted for salivation, the statistics of the General Hospital for twenty-five years, show that the mortality is actually double now what it was under the salivating treatment.—Surgeon Hare *in Edinburg Medical and Surgical Journal.*

